# Serum cholinesterase may independently predict prognosis in non-small-cell lung cancer

**DOI:** 10.1186/s12885-022-09212-0

**Published:** 2022-01-21

**Authors:** Hailiang Ran, Jie Ma, Le Cai, Hai Zhou, Zhongqin Yuan, Ying Chen, Wei Chang, Yunchao Huang, Yuanyuan Xiao

**Affiliations:** 1 Department of Epidemiology and Health Statistics, School of Public Health, Kunming Medical University, 1168 West Chunrong Road, Kunming, Yunnan China; 2 The Third Affiliated Hospital of Kunming Medical University, Kunming, Yunnan China

**Keywords:** Cholinesterase, Prognosis, Non-small-cell lung cancer, Survival analysis

## Abstract

**Background:**

Serum cholinesterase (ChE) was found to be involved in cancer initiation and progression. However, the survival association between serum ChE and non-small cell lung cancer (NSCLC) has not been extensively discussed. In the present study, we aim to elevate the role of ChE in overall survival (OS) of NSCLC patients.

**Methods:**

A total of 961 histologically confirmed NSCLC patients diagnosed between 2013 and 2018 in a provincial cancer hospital in southwestern China were retrospectively selected. Relevant information, such as histological type, clinical stage, chemotherapy, smoking status, body mass index (BMI), important serum indicators (albumin, neutrophil-to-lymphocyte ratio, ChE), date of death of the patients was extracted from the computerized hospital information system. Univariate and multivariate Cox proportional hazards models were used to determine the association between baseline serum ChE measured at the diagnosis and the OS of NSCLC patients.

**Results:**

The median of baseline ChE (7700 units/liter) was used as a cut-off to dichotomize NSCLC patients. After controlling for possible confounding factors, serum ChE at diagnosis was significantly associated with OS of NSCLC: patients with higher level of ChE were observed a better prognosis (hazard ratio, HR: 0.77, 95% CI: 0.67–0.93, *p* = 0.006). Subgroup analysis revealed significant ChE-OS association for NSCLC patients: with lower systemic inflammation level (baseline NLR < 2.95, HR: 0.71, 95% CI: 0.56–0.89, *p* = 0.003), of adenocarcinoma (HR: 0.66, 95% CI: 0.54–0.80, *p* < 0.001), in advanced stage (HR: 0.77, 95% CI: 0.66–0.92, *p* < 0.01), and received chemotherapy (HR: 0.75, 95% CI: 0.59–0.96, *p* < 0.02).

**Conclusion:**

Baseline ChE may have independent prognostic value for NSCLC patients. Longitudinal studies should be performed to corroborate this finding.

**Supplementary Information:**

The online version contains supplementary material available at 10.1186/s12885-022-09212-0.

## Background

Lung cancer is a major health threat. Although abundant studies on diagnosis, treatment, and survivorship of lung cancer had been published in the past decades, it remains one of the cancers with the highest incidence and mortality. Based on the latest global cancer data from the International Agency for Research on Cancer (IARC), lung cancer has been estimated accounted for 11.4% of the total new cancer cases worldwide and 18% of total cancer related mortalities in the year of 2020. In China, lung cancer accounted for 17.9 and 23.8% of new cancer cases and cancer related deaths in 2020 [1].

Early diagnosis and treatment for cancer patients are extremely important. However, because the lack of clinical symptoms, only a few of lung cancer patients can be diagnosed at the early stage [2]. For instance, even in the year of 2020, merely 16% of lung cancer patients were diagnosed as localized disease in the United States [3]. At the same time, lung cancer is also one of the cancers with the lowest survival rate, and the improvement in prognosis has been extremely limited for decades [1, 3]. Currently, the 5-year cumulative survival rate for all types of lung cancer is around 20%, and only 5% for distant-stage disease [2–5]. Under this circumstance, exploring potential prognostic factors is imperative for lung cancer patients, particularly for non-small-cell lung cancer (NSCLC), a predominant histological type that accounts for over 80% of lung cancer patients, and generally observed better prognosis than small-cell lung cancer (SCLC) [6, 7]. For NSCLC patients, heterogeneity in prognosis was associated with a variety of factors, such as tumor size, clinical stage of the disease, presence of pulmonary or constitutional symptoms, continued smoking, etc. [7, 8]

Since the beginning of the new millennium, the association between serum biomarker and cancer survival has gradually become one of the hotspots in the field of cancer epidemiology. Attributing to easy availability and low cost in clinical practice, blood indicators emerged as ideal prognostic indexes [9]. Recently, serum enzymes, like lactate dehydrogenase [10], alkaline phosphatase [11], gamma-glutamyltransferase, and alanine aminotransferase [12], are attracting study interest. Inside this big family, serum cholinesterase (ChE), or butyrylcholinesterase/pseudocholinesterase, is an alpha-glycoprotein, with a primary function of hydrolyzing acetylcholine and other choline esters [13, 14]. ChE is generally synthesized in the liver and released into plasma immediately. Previous evidence suggested that decreased ChE was associated with multiple diseases or conditions, like chronic liver damage, inflammation, infections, and malnutrition [13]. The latest research revealed the prognostic value of serum ChE in solid tumors: a decreased level of ChE was related to a less optimistic prognosis in several cancers regardless of hepatic involvement, such as bladder cancer [15], pancreatic cancer [14], breast cancer [16], and colorectal cancer [17]. Moreover, a published study by Pedro et al. identified a role of ChE in lung cancer biology [18]. Taken together, it is reasonable to suspect that serum ChE may also be involved in the survival of NSCLC. Nevertheless, to our best knowledge, this hypothesis has not been effectively discussed.

In this study, we intend to estimate the association between serum ChE measured at disease diagnosis and the overall survival (OS) of NSCLC by using a large sample of patients. The primary objective of our study is to evaluate the potential prognostic role of ChE in NSCLC. Besides, we also discussed the heterogeneity in this association with respect to discordant characteristics of NSCLC patients.

## Material and method

### Study design

Study subjects were retrospectively determined NSCLC patients who were histologically confirmed between January 1, 2013 and December 31, 2018, at the Third Affiliated Hospital of Kunming Medical University. This hospital is also the Provincial Cancer Hospital of Yunnan, possesses an expanded and comprehensive hospital information system (HIS), which includes the patients’ information system and the follow-up system. In the patients’ information system, all clinical practice relevant data for inpatients and outpatients are recorded and uploaded on daily basis. In the current study, we extracted the following information from the patients’ information system: sex, age at the diagnosis, ethnicity, smoking history, body mass index (BMI), comorbidities, histological type, clinical stage, serum blood indicators (ChE, albumin, neutrophil-to-lymphocyte ratio), chemotherapy. The survival information of the NSCLC patients we studied was obtained from the follow-up system. All patients included into the final analysis were a subset of NSCLC patients with the complete required information. This study was approved by the Ethics Review Board of Kunming Medical University. Because of the retrospective design, informed consents from the participants had been waived.

### Variables and definitions

The outcome of study interest was the OS. The survival interval was defined as the time from histological diagnosis date to the death date of any cause. Baseline serum ChE was measured within 7 days of the diagnosis, the median of ChE (7700 units/liter, U/L) was used as the cut-off to dichotomize study subjects. Moreover, an alternative cut-off of 8237 units/liter determined by using the X-tile version 3.6.1, was further adopted to provide reference analytical results (summarized in Supplementary materials). We also extracted baseline albumin (ALB) and neutrophil-to-lymphocyte ratio (NLR), which were commonly used nutritional status and systemic inflammation indicators, to adjust for possible confounding. For ALB, we adopted a recommended cut-off of 35 g/L [10]. NLR was dichotomized by using the median.

### Statistical analysis

Descriptive statistics were used to illustrate and compare general characteristics of the participants. The survival curves for NSCLC patients of different baseline ChE levels were drawn and compared by using Kaplan-Meier method and the log-rank test. Univariate and multivariate Cox proportional hazards models were used to evaluate the crude and adjusted associations between baseline serum ChE and the OS of NSCLC patients. Subgroup analyses based on chemotherapy, clinical stage, NLR level, and histological types were further performed. Variables that achieved a less strict significance (*p* < 0.10) in univariate analyses were included into the subsequent multivariate model. A two-tailed probability less than 0.05 was deemed statistically significant. All statistical analyses were performed in R software (Version: 3.6.2, The R Foundation for Statistical Computing, Vienna, Austria), “survival”, “ggplot2” and “survminer” packages were mainly adopted in the analytical process.

## Results

### Characteristics of study subjects

We totally retrospectively identified 1055 histologically confirmed NSCLC patients between 2013 and 2018. Ninety-four (*N* = 94) had missing values in critical variables and had been deleted. The final analysis was based on 961 patients. General characteristics of the study subjects were described in Table 1: the mean diagnosis age was 61.15 years; the majority of the patients were males (64.60%); more than half of the patients reported smoking history (60.00%); adenocarcinoma and squamous cell carcinoma combined accounted for 95.6% of all the patients. The median survival length of all patients was 374 days (Inter-quartiles range, IQR: 570 days). Patients with a higher level of baseline serum ChE (ChE > = 7700 U/L) were observed much longer median survival length than patients with lower baseline ChE (ChE < 7700 U/L, 523.39 versus 283.00 days).

**Table 1:** General characteristics of 961 NSCLC patients

Characteristics	All patients (*N* = 961)	The lower group	The higher group	*p* value
		(ChE < 7700 U/L, *N* = 482)	(ChE > = 7700 U/L, *N* = 479)	
Sex				
Female	340 (35.40)^c^	136 (28.20)^c^	204 (42.60)^c^	< 0.001
Male	621 (64.60)^c^	346 (71.80)^c^	275 (57.40)^c^	
Age at diagnosis (Years)	61.15 (10.67)^a^	63.10 (10.92)^a^	59.18 (10.04)^a^	< 0.001
Ethnicity				
Minority	89 (9.30)^c^	55 (11.40)^c^	34 (7.10)^c^	0.041
Han majority	872 (90.70)^c^	427 (88.60)^c^	445 (92.90)^c^	
Smoking				
No	384 (40.00)^c^	169 (35.10)^c^	215 (44.90)^c^	0.003
Yes	577 (60.00)^c^	313 (64.90)^c^	264 (55.10)^c^	
BMI (kg/m^2^)	23.74 (35.88)^a^	24.36 (50.63)^a^	23.14 (6.58)^a^	0.603
Chemotherapy				
No	443 (46.10)^c^	239 (49.60)^c^	204 (42.60)^c^	0.035
Yes	518 (53.90)^c^	243 (50.40)^c^	275 (57.40)^c^	
Complications				
No	521 (54.20)^c^	262 (54.40)^c^	259 (54.10)^c^	0.981
Yes	440 (45.80)^c^	220 (45.60)^c^	220 (45.90)^c^	
Histological type				
Adenocarcinoma	628 (65.30)^c^	283 (58.70)^c^	345 (72.00)^c^	< 0.001
Squamous cell carcinoma	291 (30.30)^c^	177 (36.70)^c^	114 (23.80)^c^	
Large cell carcinoma	8 (0.80)^c^	6 (1.20)^c^	2 (0.40)^c^	
Multiple types	34 (3.50)^c^	16 (3.30)^c^	18 (3.80)^c^	
Stage				
Early stage	84 (8.70)^c^	29 (6.00)^c^	55 (11.50)^c^	0.004
Advanced stage	877 (91.30)^c^	453 (94.00)^c^	424 (88.50)^c^	
Survival length (Day)	374.00 (147.00, 717.00)^b^	276.74 (107.00, 587.75)^b^	483.43 (219.00, 841.50)^b^	< 0.001
ALB (U/L)	42.50 (38.57, 45.20)^b^	39.95 (35.86, 42.99)^b^	44.19 (42.10, 46.60)^b^	< 0.001
NLR (Unit free)	2.95 (1.97, 4.36)^b^	3.43 (2.22, 5.20)^b^	2.53 (1.83, 3.46)^b^	< 0.001
ChE (U/L)	7700.00 (6287.00, 8900.00)^b^	–	–	

^a^Mean with standard deviation (SD)

^b^Median with interquartile range (IQR)

^c^Frequency with proportion (%)

### Baseline serum ChE and the OS of NSCLC

**Fig. 1:**
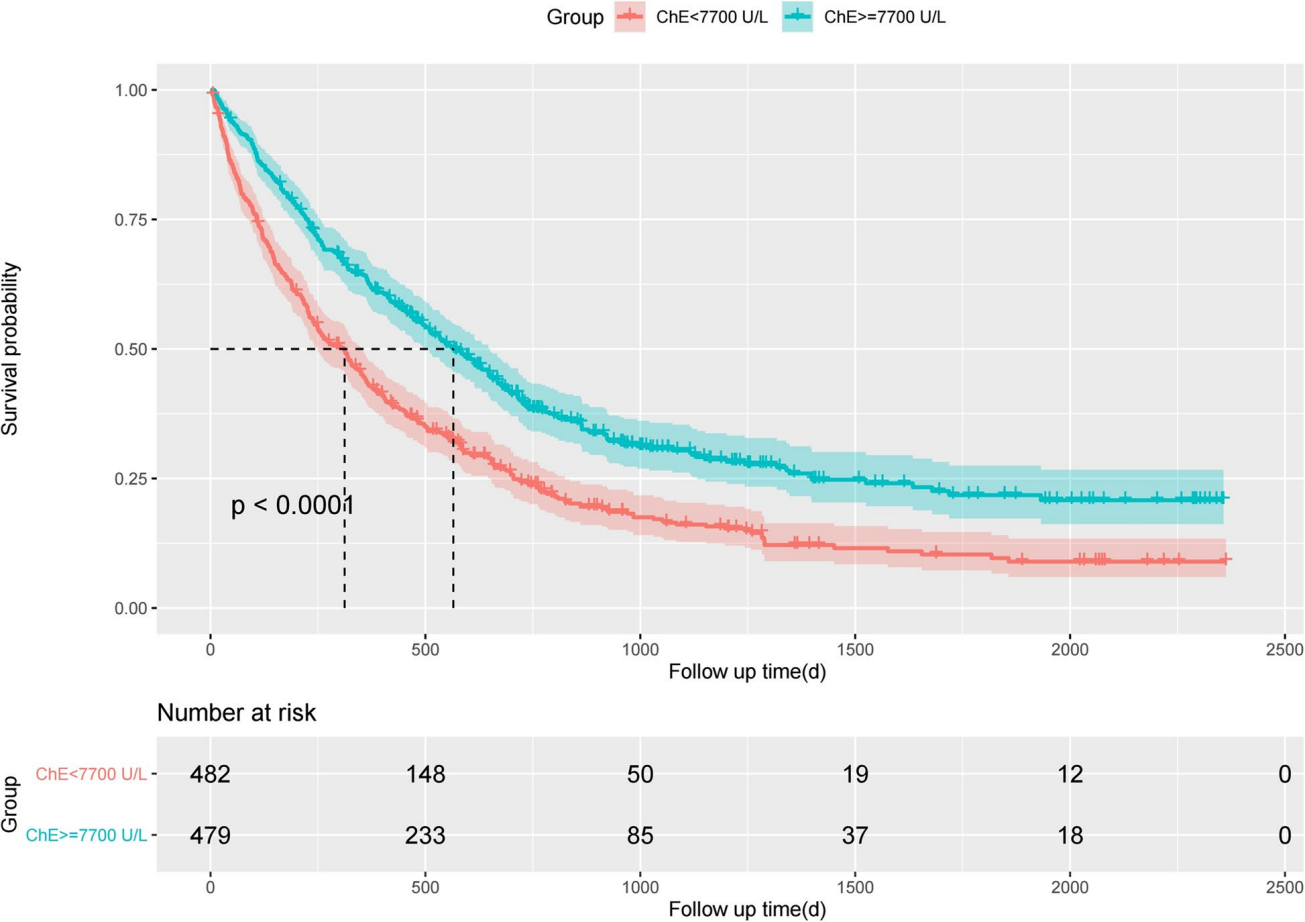
Kaplan-Meier survival curves for NSCLC patients with different baseline serum ChE levels

Figure 1 presents an overview of the survival outcome for NSCLC patients with higher and lower baseline serum ChE. OS of the higher ChE group was significantly superior to the lower ChE group (log-rank test statistic: 43.2, *p* = 10^− 11^). Univariate and multivariate Cox proportional hazards models fitting results were shown in Table 2. Univariate models identified 8 potential covariates: sex, age at diagnosis, smoking, chemotherapy, histological type, clinical stage, baseline serum ALB and NLR. After adjustment by using the multivariate model, baseline serum ChE remained as a significant prognostic factor: compared with NSCLC patients of lower baseline ChE, the hazard ratio (HR) for NSCLC patients with higher level of ChE was 0.77 (95% CI: 0.67–0.93, *p* = 0.006).

**Table 2:** Univariate and multivariate Cox proportional hazards model results

Covariates	Univariate Cox model		Multivariate Cox model	
	Crude HR (90% CI)	*p* value	Adjusted HR (95% CI)	*p* value
Sex (Male)	1.63 (1.42, 1.86)	< 0.001	1.32 (1.07, 1.64)	0.01
Age at diagnosis (+ 5 years)	1.08 (1.05, 1.12)	< 0.001		
Smoking (Yes)	1.31 (1.15, 1.49)	< 0.001		
BMI (+ 1)	1.00 (0.99, 1.01)	0.21		
Chemotherapy (Yes)	0.60 (0.53, 0.68)	< 0.001	0.55 (0.47, 0.64)	< 0.001
Comorbidities (Yes)	0.95 (0.85, 1.09)	0.57		
Histological type				
Squamous cell carcinoma	1.36 (1.19, 1.55)	< 0.001		
Large cell carcinoma	0.82 (0.39, 1.71)	0.65		
Multiple types	1.76 (1.28, 2.41)	0.003	1.77 (1.20, 2.61)	0.017
Stage (Advanced stage)	4.95 (3.40, 7.21)	< 0.001	4.78 (3.18, 7.18)	< 0.001
Baseline serum ALB (> = 35 U/L)	0.40 (0.33, 0.48)	< 0.001	0.53 (0.42, 0.68)	< 0.001
Baseline serum NLR (+ 5)	1.34 (1.28, 1.40)	< 0.001	1.25 (1.17, 1.34)	< 0.001
Baseline serum ChE (> = 7700 U/L)	0.61 (0.53, 0.69)	< 0.001	0.77 (0.67, 0.93)	0.006

**Fig. 2:**
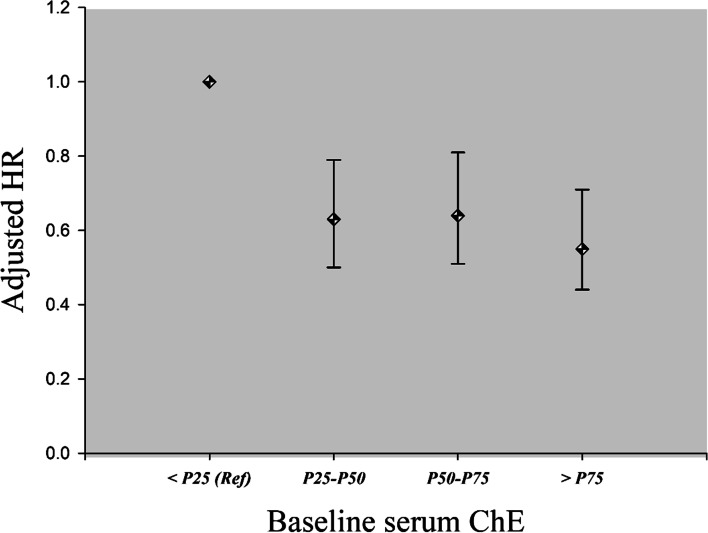
Dose-response association between baseline serum ChE and the OS of NSCLC

To verify reliability and the trend of this association, we further divided NSCLC patients into 4 groups based on the quartiles of baseline serum ChE: group 1 (ChE < 6287 U/L), group 2 (6287 U/L < = ChE < 7700 U/L), group 3 (7700 U/L < = ChE < 8900 U/L), group 4 (ChE > = 8900 U/L). By taking group 1 as the reference group, after adjusted for potential covariates identified in the previous univariate models, we found that the adjusted HRs for group 2 to group 4 were 0.63 (95% CI: 0.50–0.79, *p* < 0.001), 0.64 (95% CI: 0.51–0.81, *p* < 0.001) and 0.55 (95% CI: 0.43–0.71, *p* < 0.001), respectively (Fig. 2). However, the trend for dose-response association was weak.

### Subgroup analysis

**Fig. 3:**
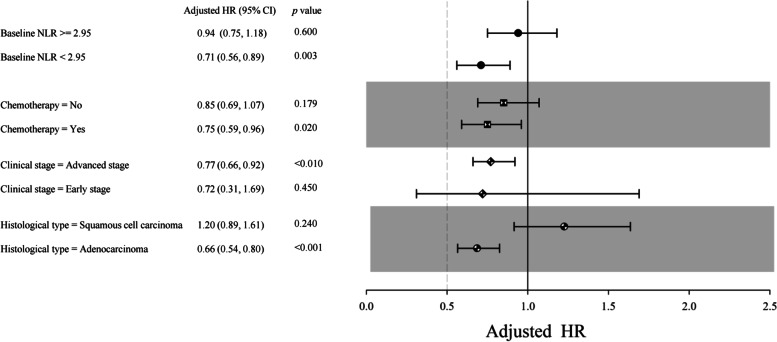
Subgroup analysis results by NLR, chemotherapy, stage, and histological type

We further performed a series of subgroup analyses based on several important characteristics of the patients: baseline serum NLR, chemotherapy, clinical stage, and histological type. The significant ChE-OS association was found only in patients: with lower baseline serum NLR level (NLR < 2.95, HR: 0.71, 95% CI: 0.56–0.89, *p* = 0.003), diagnosed of adenocarcinoma (HR: 0.66, 95% CI: 0.54–0.80, *p* < 0.001), in advanced clinical stage (HR: 0.77, 95% CI: 0.66–0.92, *p* < 0.01), and received chemotherapy (HR: 0.75, 95% CI: 0.59–0.96, *p* = 0.02) (Fig. 3).

## Discussion

In the current retrospective study, we examined the prognostic role of serum ChE measured at the diagnosis in the OS of 961 NSCLC patients. We found that baseline ChE was significantly associated with the OS of NSCLC patients: a higher level of serum ChE was related to a 23% reduction in death hazard. Besides, discordant ChE-OS associations have been identified for NSCLC patients of different characteristics. These major findings suggest that ChE would be a potential prognostic indicator for NSCLC.

The positive association between ChE and other malignancies other than NSCLC has also been reported previously. For instance, a newly published study by Vartolomei et al. found that ChE was associated with the OS of patients with salvage radical prostatectomy [19], another study by Kimura et al. observed that decreased ChE level was associated with poorer recurrence-free survival (RFS) in patients with non-muscle invasive bladder cancer (NMIBC) [15]. Normally, ChE is an indicator for acute and chronic liver damage. However, recent studies suggested that ChE can be involved in the process of cancer [15, 20]. Particularly, decreased ChE activity and the resultant accumulation of choline in cancer tissue could lead to cholinergic over-stimulation, which can promote cell growth in lung carcinoma [18, 21–24]. However, the scarcity of available studies stresses the necessity in untangling the underlying mechanism behind this association.

Further performed subgroup analysis revealed a prominent association between ChE and OS only for lung adenocarcinoma patients. A possible suspicion behind this finding could be that ChE activity may vary across histological types of NSCLC. In fact, laboratory studies have already confirmed that for different types of carcinoma cells, the expression of ChE tends to be different [25]. Moreover, declined ChE activity has been observed in squamous cell carcinoma, which may suggest that the sensitivity for ChE is poor in this type of lung cancer [18]. Therefore, it would be interesting to investigate survival benefit of ChE enhancement therapy among lung adenocarcinoma patients in future studies. A previously published experimental study had already observed that enhancing ChE expression could suppress liver carcinoma cell development and tumorigenicity, both in vitro and in vivo [26]. It is also possible that this identified association in adenocarcinoma may attribute to molecular target therapy [7]. However, as this part of therapeutic information was unavailable in the current study, this hypothesis invites further investigation.

Another very important finding of our research is that, when dichotomizing NSCLC patients by using the median of NLR, the association between ChE and OS was significant only in patients of lower NLR. The most plausible explanation behind this phenomenon would be that NLR is a sensitive indicator for systemic inflammation, and it has been repeatedly verified that systemic inflammation is associated with deteriorated survival of cancer patients [27, 28]. Therefore, for NSCLC patients with elevated NLR, the unfavorable influence of systemic inflammation on OS may offset the survival benefit of ChE. Another possible reason is that inflammation could directly influence bioactivity of ChE. It had been observed that the level of ChE was inhibited within the environment of high inflammation, and recovered along with the alleviation of inflammation [29]. This finding may suggest that, for NSCLC patients with high systemic inflammation level, simply enhance ChE can be futile in improving survival.

Existing research suggests that expression of cholinesterase is associated with response to chemotherapy in cancer patients [30]. For example, lower ChE level may predict a poorer prognosis for biliary tract cancer patients received chemotherapy of gemcitabine and cisplatin [31]. Another newly published study revealed the potential value of ChE as a real-time indicator of chemotherapy performance [30]. A reasonable explanation behind this result could be that ChE was associated with multiple conditions, particularly liver function and malnutrition which are generally related to chemotherapy [32]. Our finding may suggest that serum ChE level should be take into consideration prior to the initiation of chemotherapy. For clinical stage of NSCLC, although we only identified a prominent ChE-OS association in advanced NSCLC patients, considering the wide confidence interval caused by small sample size, whether the significant association also exists for early stage NSCLC patients remains debatable, and should be further discussed by future study with larger sample size.

All the above findings suggest the potential role of ChE in NSCLC survival. Monitoring the variation of ChE might be of prognostic significance. The regulation of ChE has shown promising effect in therapeutic strategy of cancer [33]. A previously published animal study observed that the progress of gastric cancer was significantly inhibited by upregulating cholinergic expression in mice [34]. Besides, a combination of chemotherapy and upregulated ChE activity also is a potential anticancer treatment [35]. Nevertheless, this treatment still confronts many challenges ahead before can be used clinically.

Although our study was among the first to exhaustively investigate the prognostic role of ChE in NSCLC patients by using a large sample, several limitations should be pointed out. First, the study design was retrospective, and the included subjects were chosen exclusively from a single Chinese cancer institution, therefore the generalization of study results should be made with caution. Prospective multi-center studies should be conducted. Second, although we have successfully adjusted for some important factors when estimating the adjusted association between ChE and OS of NSCLC, the information of some other potential confounding factors was unavailable, like tumor location, tumor size, smoking exposure level, immunohistochemistry of PD-L1 protein, molecular profiling, molecular target therapy, the risk of confounding bias cannot be precluded.

## Conclusion

In this retrospective study, decreased baseline ChE was associated with less optimistic OS of NSCLC patients. Moreover, systemic inflammation level, histological type, clinical stage, and chemotherapy may influence this ChE-OS association. Future longitudinal studies with more representative sample of NSCLC patients should be conducted to corroborate our findings.

## Supplementary Information

**Additional file 1.**

## Data Availability

The datasets analyzed for the current study are available from the corresponding author under reasonable request.

## Abbreviations

ALB: Albumin
BMI: Body mass index
ChE: Cholinesterase
HR: Hazard ratio
IARC: International Agency for Research on Cancer
NLR: Neutrophil-to-lymphocyte ratio
NSCLC: Non-small cell lung cancer
OS: Overall survival
