# Supplementary material for: Serum cholinesterase may independently predict prognosis in non-small-cell lung cancer
Source: BMC Cancer. 2022 Jan 21;22:93. doi: 10.1186/s12885-022-09212-0 (PMC8783506; doi:10.1186/s12885-022-09212-0)
Supplement: Supplementary file 1 — Additional file 1. [file 12885_2022_9212_MOESM1_ESM.docx]

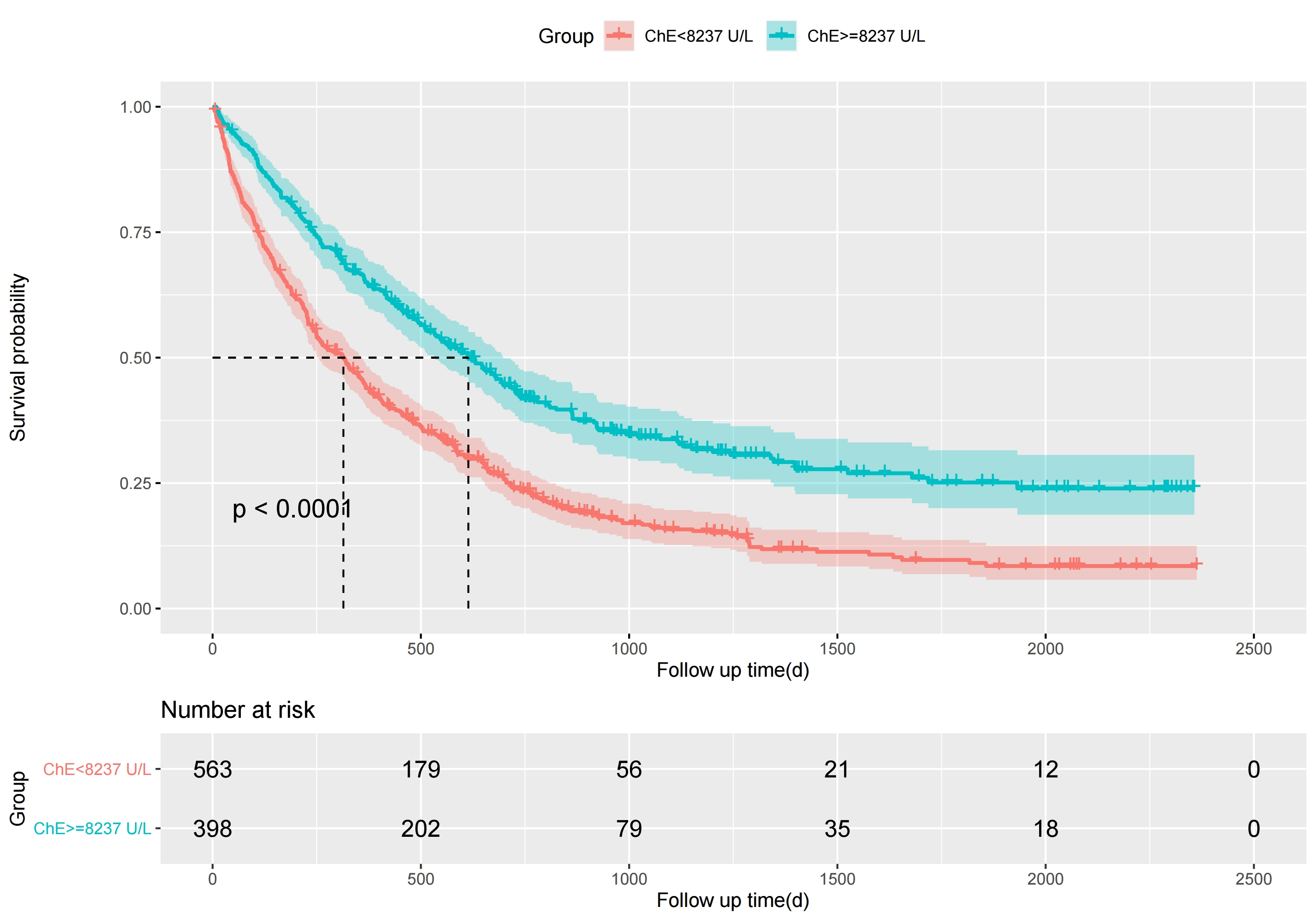


Figure S1 Kaplan-Meier survival curves for NSCLC patients with different baseline serum ChE levels.


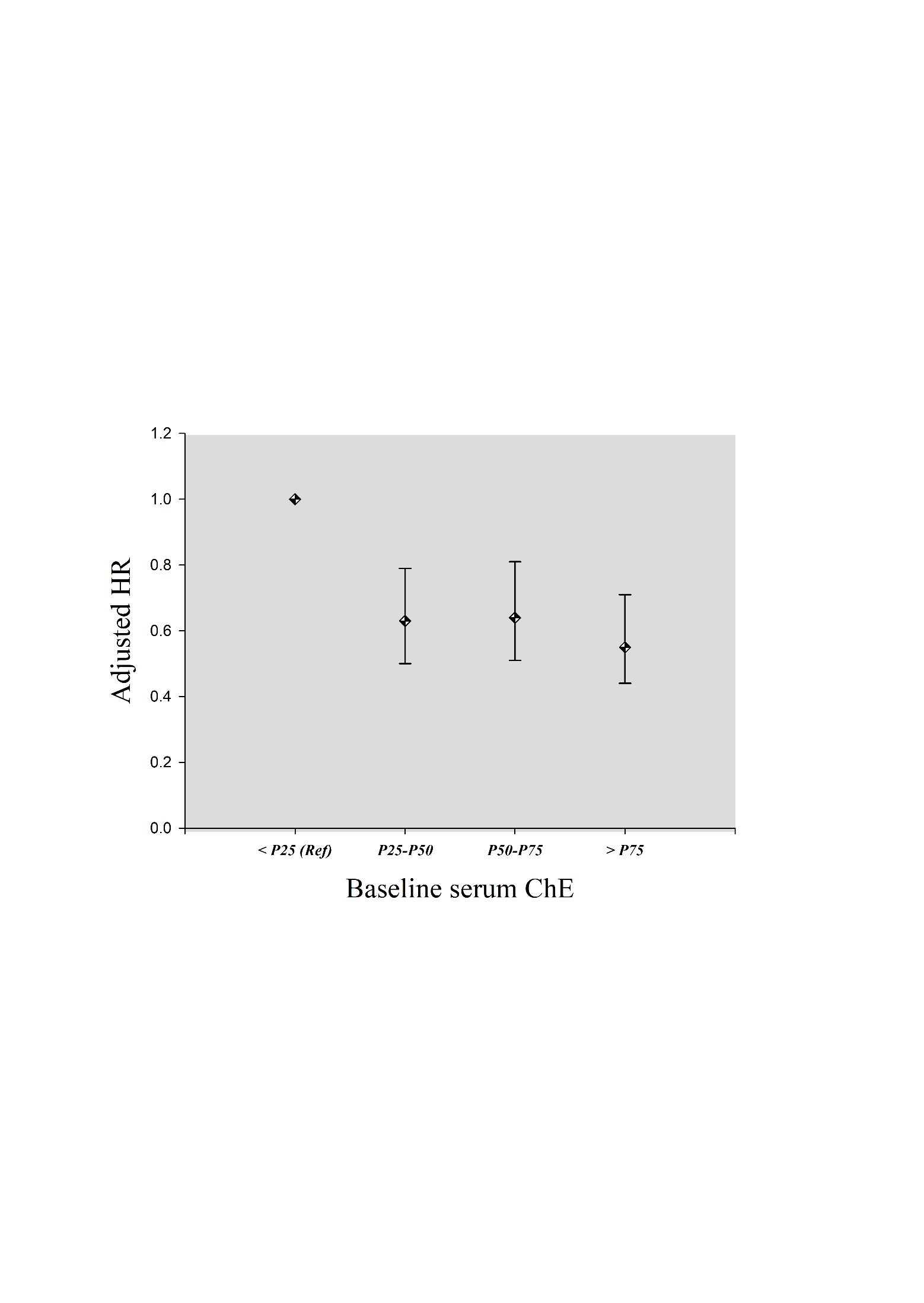


Figure S2 Dose-response association between baseline serum ChE and the OS of NSCLC


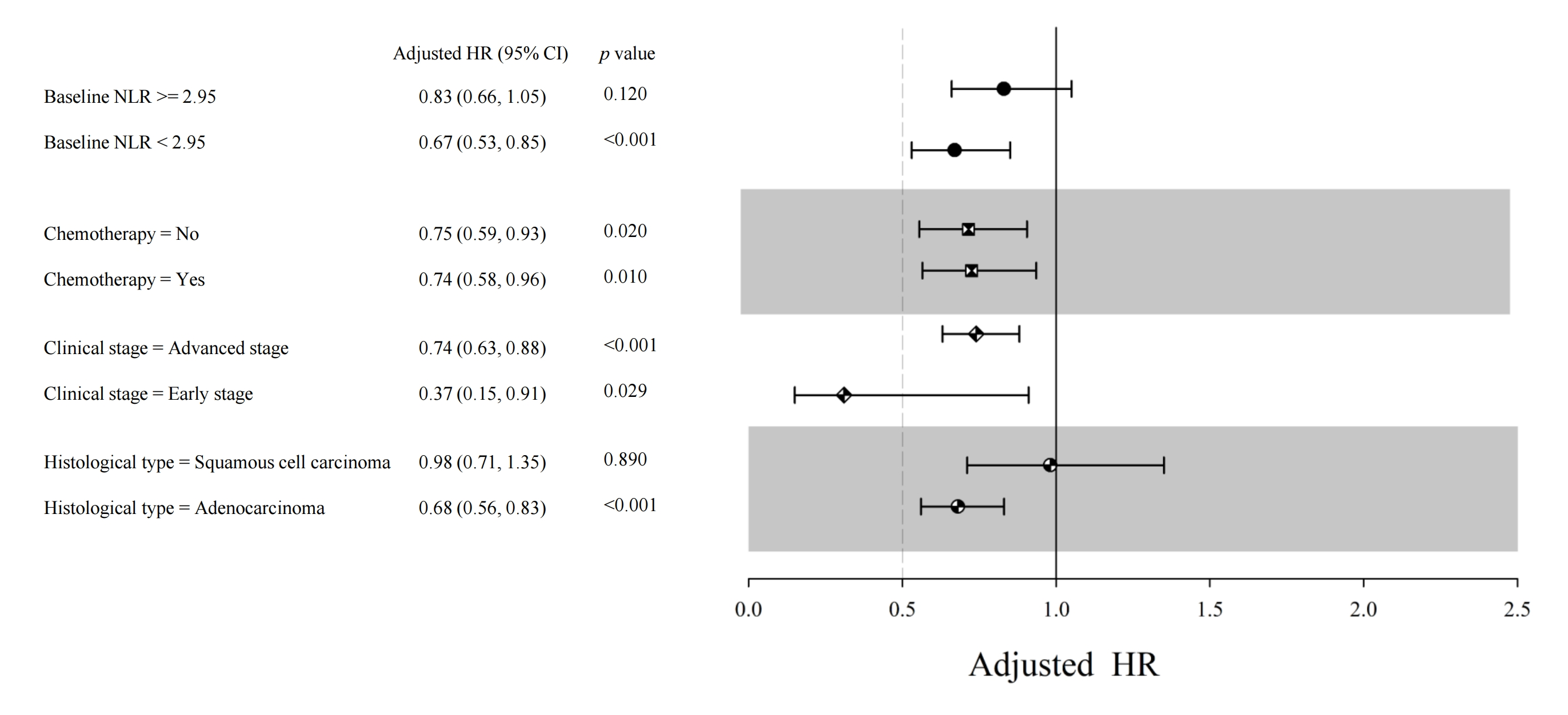


Figure S3 Subgroup analysis results by NLR, chemotherapy, stage, and pathological type

**Table S1 General characteristics of 961 NSCLC patients**

| **Characteristics** | **All patients (*N*=961)** | **The lower group** | **The higher group** | ***p* value** |
| --- | --- | --- | --- | --- |
|  |  | **(ChE < 8237 U/L, *N*=586)** | **(ChE > = 8237 U/L, *N*=375)** |  |
| Sex |  |  |  |  |
| Female | 340 (35.40)^c^ | 165 (28.20)^c^ | 175 (46.70)^c^ | <0.001 |
| Male | 621 (64.60)^c^ | 421 (71.80)^c^ | 200 (53.30)^c^ |  |
| Age at diagnosis (Years) | 61.15 (10.67)^a^ | 62.56 (10.79)^a^ | 58.94 (10.10)^a^ | <0.001 |
| Ethnicity |  |  |  |  |
| Any minority | 89 (9.30)^c^ | 63 (10.80)^c^ | 26 (6.90)^c^ | 0.114 |
| Han majority | 872 (90.70)^c^ | 523 (89.2)^c^ | 349 (93.10)^c^ |  |
| Smoking |  |  |  |  |
| No | 384 (40.00)^c^ | 202 (34.50)^c^ | 182 (48.50)^c^ | <0.001 |
| Yes | 577 (60.00)^c^ | 384 (65.60)^c^ | 193 (51.50)^c^ |  |
| BMI (kg/m^2^) | 23.74 (35.88)^a^ | 24.01 (45.80)^a^ | 23.33 (7.24)^a^ | 0.774 |
| Chemotherapy |  |  |  |  |
| No | 443 (46.10)^c^ | 290 (49.50)^c^ | 153 (40.80)^c^ | 0.010 |
| Yes | 518 (53.90)^c^ | 296 (50.50)^c^ | 222 (59.20)^c^ |  |
| Comorbidity |  |  |  |  |
| No | 521 (54.20)^c^ | 320 (54.60)^c^ | 201 (53.60)^c^ | 0.811 |
| Yes | 440 (45.80)^c^ | 266 (45.40)^c^ | 174 (46.40)^c^ |  |
| Pathological type |  |  |  |  |
| Adenocarcinoma | 628 (65.3)^c^ | 341 (58.2)^c^ | 287 (76.50)^c^ | <0.001 |
| Squamous cell carcinoma | 291 (30.3)^c^ | 213 (36.3)^c^ | 78 (20.80)^c^ |  |
| Large cell carcinoma | 8 (0.80)^c^ | 6 (1.00)^c^ | 2 (0.50)^c^ |  |
| Multiple types | 34 (3.50)^c^ | 26 (4.40)^c^ | 8 (2.10)^c^ |  |
| Cancer stage |  |  |  |  |
| Early | 84 (8.70)^c^ | 39 (6.70)^c^ | 45 (12.00)^c^ | 0.006 |
| Advanced | 877 (91.30)^c^ | 547 (93.30)^c^ | 330 (88.00)^c^ |  |
| Survival length (Day) | 374.00 (147.00, 717.00)^b^ | 283.00 (111.00, 613.92)^b^ | 523.39 (246.50, 871.50)^b^ | <0.001 |
| ALB (U/L) | 42.50 (38.57, 45.20)^b^ | 40.88 (36.39, 43.65)^b^ | 44.73 (42.38, 47.01)^b^ | <0.001 |
| NLR (Unit free) | 2.95 (1.97, 4.36)^b^ | 3.32 (2.22, 5.06)^b^ | 2.37 (1.76, 3.30)^b^ | <0.001 |
| ChE (U/L) | 7700.00 (6287.00, 8900.00)^b^ | - | - |  |
| ^a^Mean with standard deviation (SD) | | | | |
| ^b^Median with interquartile range (IQR) | | | | |
| ^c^Frequency with proportion (%) | | | | |

**Table S2 Univariate and multivariate Cox proportional hazards model results**

| **Covariates** | **Univariate Cox model** | |  | **Multivariate Cox model** | |
| --- | --- | --- | --- | --- | --- |
|  | **Crude HR (90% CI)** | ***p* value** |  | **Adjusted HR (95% CI)** | ***p* value** |
| Sex (Male) | 1.63 (1.42, 1.86) | <0.001 |  | 1.33 (1.07, 1.65) | 0.010 |
| Age at diagnosis (+5 years) | 1.08 (1.05, 1.12) | <0.001 |  |  |  |
| Smoking (Yes) | 1.31 (1.15, 1.49) | <0.001 |  |  |  |
| BMI (+1) | 1.00 (0.99, 1.01) | 0.21 |  |  |  |
| Chemotherapy (Yes) | 0.60 (0.53, 0.68) | <0.001 |  | 0.55 (0.47, 0.64) | <0.001 |
| Comorbidities (Yes) | 0.95 (0.85, 1.09) | 0.57 |  |  |  |
| Pathological type |  |  |  |  |  |
| Squamous cell carcinoma | 1.36 (1.19, 1.55) | <0.001 |  |  |  |
| Large cell carcinoma | 0.82 (0.39, 1.71) | 0.65 |  |  |  |
| Multiple types | 1.76 (1.28, 2.41) | 0.003 |  | 1.61 (1.09, 2.38) | 0.017 |
| Stage (Advanced stage) | 4.95 (3.40, 7.21) | <0.001 |  | 4.76 (3.17, 7.15) | <0.001 |
| Baseline serum ALB (>= 35 U/L) | 0.40 (0.33, 0.48) | <0.001 |  | 0.54 (0.42, 0.68) | <0.001 |
| Baseline serum NLR (+5) | 1.34 (1.28, 1.40) | <0.001 |  | 1.25 (1.17, 1.33) | <0.001 |
| Baseline serum ChE (>= 8237 U/L) | 0.57 (0.50, 0.65) | <0.001 |  | 0.75 (0.63, 0.88) | <0.001 |
